# Definition and validation of recompensation in patients with primary biliary cholangitis-related decompensated cirrhosis treated with ursodeoxycholic acid: Based on the BAVENO VII criteria

**DOI:** 10.1515/jtim-2025-0034

**Published:** 2025-07-31

**Authors:** Dawei Ding, Lina Cui, Gui Jia, Boling Wang, Linhua Zheng, Juan Deng, Ruiqing Sun, Xiufang Wang, Yulong Shang, Guanya Guo, Ying Han

**Affiliations:** State Key Laboratory of Holistic Integrative Management of Gastrointestinal Cancers, National Clinical Research Center for Digestive Diseases, Xijing Hospital, The Air Force Military Medical University, Xi'an, Shaanxi Province, China

**Keywords:** recompensation, cirrhosis, model for end-stage liver disease, cholestasis

## Abstract

**Background and Objectives:**

Few studies have provided real-world data on the biochemical response, risk assessment, and prognosis of patients with primary biliary cholangitis (PBC)-related decompensated cirrhosis undergoing ursodeoxycholic acid therapy. The objective of this study is to define recompensation in this patient population based on the BAVENO VII criteria.

**Methods:**

This retrospective analysis included 170 patients with cirrhosis who presented with ascites, hepatic encephalopathy, and/or variceal bleeding as their initial decompensating events at Xijing Hospital from 2006 to 2023. Events of further decompensation, liver transplantation, and liver-related death were recorded.

**Results:**

Alkaline phosphatase (ALP) had complex prognostic value in patients with PBC-related decompensated cirrhosis receiving ursodeoxycholic acid therapy. In patients with normal total bilirubin (TBIL) at the 1-year follow-up, elevated ALP was associated with poor prognosis (hazard ratio [HR]: 2.57, 95% confidence interval [CI]: 1.12-5.87, *P* = 0.025), whereas in those with elevated TBIL, decreased ALP was associated with poor prognosis (HR: 0.53, 95% CI: 0.26-1.08, *P* = 0.082). A Model for End-Stage Liver Disease score < 10 and the absence of decompensating events from the last decompensated state over the next 12 months were used to assess PBC recompensation. During follow-up, 26% (45/170) of patients experienced at least one episode of recompensation. Compared with observations in the non-recompensation group, the recompensation group exhibited a longer liver transplantation-free survival (HR: 16.48, 95% CI: 2.23-121.57, *P* = 0.006), lower rates of further decompensation (22% *vs*. 63%, *P* < 0.001), a significant reduction in high-risk patients (*P* < 0.05, all), and notable improvements in serum indicators (platelet count, TBIL, albumin, and international normalized ratio). Baseline platelet and TBIL levels, the 1-year Rotterdam criteria, and severe interface hepatitis were associated with recompensation.

**Conclusions:**

We defined PBC recompensation as a Model for End-Stage Liver Disease score < 10 and the absence of decompensating events from the last decompensated state for the next 12 months, aligned with the requirements of BAVENO VII for patients with PBC-related decompensated cirrhosis.

## Introduction

Primary biliary cholangitis (PBC) is a chronic autoimmune liver disease characterized by progressive, non-suppurative, destructive inflammation of the small bile ducts within the liver, leading to intrahepatic cholestasis.^[1, 2, 3]^ Epidemiologically, PBC affects approximately 14.6 per 100,000 individuals globally, with significant geographic variability (including high prevalence in Northern Europe and North America).^[4]^ Notably, the 5-year incidence of decompensated liver disease has been estimated at 15% in a large community-based study of 770 patients in northeast England.^[5]^

Current management relies heavily on the administration of ursodeoxycholic acid (UDCA). However, up to 30%-40% of patients exhibit a suboptimal response to UDCA.^[6, 7, 8]^ Critically, emerging evidence suggests that the efficacy of UDCA diminishes in decompensated cirrhosis. Furthermore, existing “biochemical response” criteria (including Paris-I/II) have never been validated in this population, raising concerns about the accuracy of risk stratification in advanced disease. Therapeutic options for patients with decompensated PBC remain alarmingly limited: obeticholic acid, despite its U. S. Food and Drug Administration approval, is contraindicated in patients with Child-Pugh B/C cirrhosis because of the risk of fatal hepatotoxicity. Fibrates (including bezafibrate) may paradoxically worsen pruritus and cholestasis in these patients.^[9,10]^

The most recent BAVENO VII publication^[11]^ proposed a consensus definition of recompensation, which requires the fulfillment of all three criteria: (A) removal, suppression, or cure of the primary etiology of cirrhosis; (B) resolution of ascites (off diuretics), encephalopathy (off lactulose/rifaximin), and absence of recurrent variceal hemorrhage (for at least 12 months); (C) stable improvement of liver function tests (albumin [ALB], international normalized ratio, bilirubin), providing a prognostic framework for assessing patients with decompensated PBC receiving UDCA therapy. However, these criteria need to be further clarified for PBC, particularly regarding the first criterion, which defines the suppression of the primary etiology of cirrhosis.

Therefore, in the present study, we explored recompensation criteria suitable for patients with PBC who had ascites, hepatic encephalopathy, and/or variceal bleeding as their first decompensating event, based on the BAVENO VII criteria.

## Materials and methods

### Study population

We analyzed 170 consecutive patients with PBC-related decompensated cirrhosis who were diagnosed and treated at the Xijing Hospital of Digestive Diseases (Xi’an, Shaanxi, China) from June 2006 to October 2023. PBC was diagnosed according to the European Association for the Study of the Liver.^[12]^

The inclusion criteria were as follows: (1) standardized UDCA treatment; (2) meeting the imaging, radiological, or histological diagnostic criteria for cirrhosis; and (3) presence of ascites, hepatic encephalopathy, and/or variceal bleeding as the first presentation of decompensation.

The exclusion criteria were: (1) comorbid viral hepatitis, alcohol-related liver disease, drug-induced liver injury, autoimmune hepatitis, metabolic dysfunction-associated fatty liver disease, or hereditary liver disease; (2) follow-up time of less than 1 year; (3) treatment with glucocorticoids; (4) comorbid malignant tumors (excluding tumors that had been cured) or prior liver transplantation; and (5) dysfunction of vital organs, such as the heart, kidneys, and brain.

### Study design

This was a retrospective cohort study. First, we identified the risk factors associated with liver transplant (LT)— free survival and defined recompensation criteria for PBC-related decompensated cirrhosis. Second, we evaluated the recompensation status in this group of patients treated with UDCA and analyzed the relation between recompensation and prognosis. Third, we explored dynamic changes in risk stratification and serum biochemical indicators between the recompensation and non-recompensation groups. Finally, we explored the risk factors associated with recompensation. The GLOBE risk score was used to estimate risk stratification and was calculated at baseline and in the first, second, and third years of follow-up.^[13]^ The primary outcome was liver-related death or LT. The secondary outcome was further decompensation, defined according to the BAVENO VII criteria,^[11]^ and jaundice was defined as total bilirubin (TBIL) > 3 mg/dL.^[14]^ Biochemical response was assessed using Paris I,^[6]^ Paris II,^[15]^ Rotterdam, ^[16]^ and Toronto^[17]^ at 1 year after enrollment. Separate retrospective analyses were performed using different response criteria. The study design was approved by the Ethics Committee of Xijing Hospital of the Air Force Military Medical University.

### Treatment and follow-up

The baseline date was defined as the date on which the first decompensated event occurred during follow-up at our center. All patients with decompensated PBC received UDCA monotherapy at a standardized dose of 13 to 15 mg/kg/d. No escalation to second-line therapies was pursued owing to the high risk of adverse events associated with decompensated cirrhosis. Instead, these patients underwent intensified monitoring for disease progression (including the model for end-stage liver disease [MELD] score tracking and hepatic function tests every 3 months). Cirrhosis treatment was based on the standard of care according to the available recommendations at the time of accrual/follow-up. Concurrent medications were limited to the management of complications (including ascites and hepatic encephalopathy), with no immunomodulators or other hepatoprotective agents used.

Follow-up visits were based on clinical and laboratory assessments every 6-12 months or earlier (depending on clinical status), ultrasonography (screening for ascites) every 6-12 months, and upper endoscopy every 2-3 years in variance-free patients. Liver biopsies were analyzed according to the METAVIR scoring system^[18]^ and evaluated by two qualified and experienced pathologists who were blinded to the serological test results. Patients were classified as lost to follow-up if they missed three consecutive scheduled visits without contact. Patients lost to follow-up were excluded.

### Statistical analysis

SPSS (version 26.0; IBM Corp., Armonk, NY, USA) and R statistical software 4.13 (Tsinghua) were used. The Shapiro–Wilk test was used to determine whether the data were normally distributed. Normally distributed continuous variables were expressed as mean ± standard deviation, and non-normally distributed variables were expressed as medians (interquartile range, IQR). Comparisons of the means of two continuous normally distributed variables were performed using the independent samples Student’s *t*-test, and the medians of two continuous non-normally distributed variables were compared using the Mann– Whitney *U* test. Cox regression analyses were used to examine the relation between prognostic indicators and LT-free survival, as well as the risk factors for recompensation. All variables with statistical significance in the univariate analysis were adjusted in the multivariate model. The DeLong test and C-index were used to compare the areas under the receiver operating characteristic curves. Statistical significance was set at *P* < 0.05.

## Generalized conclusions from experimental data

### Patient enrollment and follow-up characteristics

**Figure 1:**
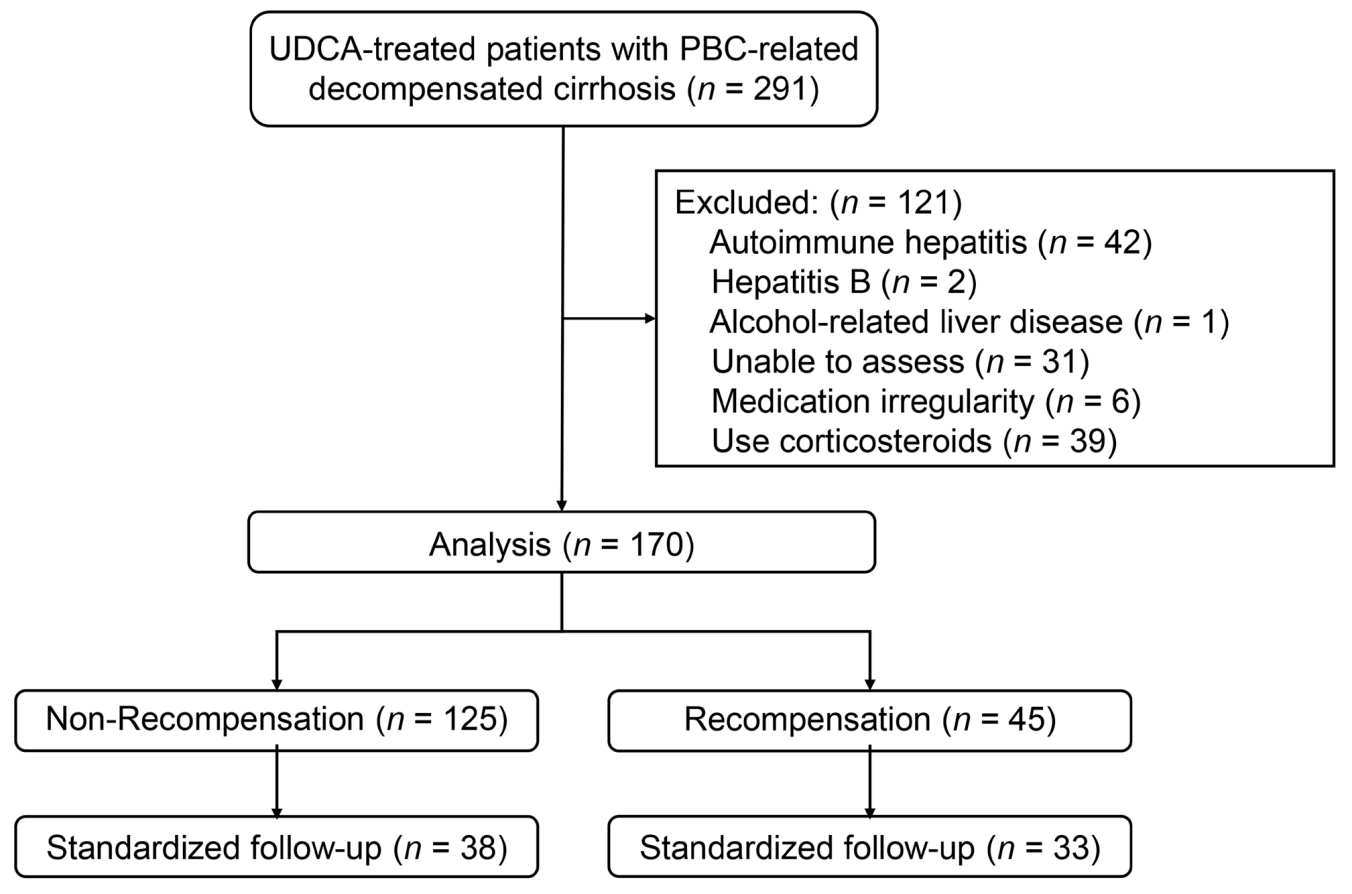
Case screening process and study design. Recompensation was defined as the absence of decompensating events for 12 months following the last decompensated state and MELD <10. Standardized follow-up indicates that patients had 1-, 2-, and 3-year follow-up data available simultaneously. UDCA: ursodeoxycholic acid; PBC: primary biliary cholangitis; MELD: model for end-stage liver disease.

Figure 1 presents a flowchart of this study. A total of 291 patients with PBC-related decompensated cirrhosis were screened. After excluding 121 patients for various reasons, 170 were enrolled for a median follow-up of 39 months (IQR, 24-68 months), of whom 151 (81%) had ascites, one (1%) had hepatic encephalopathy, 18 (11%) had variceal bleeding, and 14 (8%) had both ascites and variceal bleeding at baseline. Patients underwent UDCA for a median of 26 months (IQR, 6-36 months) before enrolment.

The baseline, 1-year, and outcome characteristics of the patients are presented in Table 1. Their mean age was 57 ± 9 years, with 89% (151/170) being women and 85% (144/170) being anti-mitochondrial antibody-positive. The median platelet (PLT), alkaline phosphatase (ALP), aspartate aminotransferase (AST), TBIL, and ALB values were 0.72 × lower limit of normal (LLN), 1.52 × upper limit of normal (ULN), 1.43 × ULN, 1.09 × ULN, and 0.91 × LLN. The median MELD and GLOBE scores were 12 (IQR, 10-14) and 1.40 (IQR, 0.84-2.09), respectively. The included patients exhibited low biochemical response rates, with only 48% (82/170) achieving a response according to the Paris-1 criteria. During follow-up, 28% (16/170) of the patients experienced primary outcomes, and 52% (89/170) experienced secondary outcomes.

**Table 1:** Baseline, 1-year, and outcome characteristics of 170 patients with PBC-related decompensated cirrhosis

Parameters	
Baseline parameters	
Age (years)	57 ± 9
Female (*n*, %)	151 (89)
Follow-up time (months)	39 (24-68)
ALP × ULN	1.52 (0.96-2.47)
GGT × ULN	2.52 (1.29-4.99)
AMA (*n*, %)	144 (85)
PLT × LLN	0.72 (0.57-1.03)
ALT × ULN	0.96 (0.58-1.48)
AST × ULN	1.43 (1.06-2.08)
ALB × LLN	0.91 (0.83-1.00)
TBIL × ULN	1.09 (0.75-1.70)
Scr × ULN	0.69 (0.58-0.83)
IgM × ULN	0.98 (0.67-1.49)
INR	1.06 (1.00-1.16)
MELD scores	12 (10-14)
1-year parameters	
GLOBE scores	1.40 (0.84-2.09)
Paris 1 (*n*, %)	82 (48)
Paris 2 (*n*, %)	57 (34)
Rotterdam (*n*, %)	42 (25)
Toronto (*n*, %)	119 (70)
Outcomes (*n*, %)	
Primary outcome	28 (16)
Liver-related death	25 (89)
Liver transplantation	3 (11)
Secondary outcome	
Further decompensation	89 (52)
Ascites^#^	64 (72)
Variceal bleeding^#^	12 (13)
Hepatic encephalopathy^#^	1 (1)
Ascites + variceal bleeding^#^	12 (13)

Continuous variables were expressed as mean ± SD or median (IQR), while categorical variables were presented as *n* (%) 167 (98) had Scr data, 163 (96) had INR data, 151 (89) had IgM data. ^#^Clinical presentation of first decompensation events. ALP: alkaline phosphatase; GGT: gamma-glutamyl transpeptidase; AMA: anti-mitochondrial antibody; PLT: platelet; ALT: alanine aminotransferase; AST: aspartate aminotransferase; ALB: albumin; TBIL: total bilirubin; Scr: serum creatinine; IgM: Immunoglobulin M; INR: international normalised ratio; MELD: model for end-stage liver disease; ULN: upper limit of normal; LLN: lower limit of normal; IQR: interquartile range.

### Prognostic analysis at baseline and 1-year follow-up

At baseline, univariate analysis revealed that cholestasis indicators, such as ALP and gamma-glutamyl transpeptidase, were not associated with LT-free survival. Multivariate analysis demonstrated that age (hazard ratio [HR], 1.063; 95% confidence interval [CI], 1.017-1.112; *P* = 0.007), ALB level (HR, 0.009; 95% CI, 0.001-0.175; *P* = 0.002), and TBIL level (HR, 1.503; 95% CI, 1.191-1.898; *P* = 0.001) were associated with LT-free survival (Supplementary Table S1).

At the 1-year follow-up, univariate analysis showed that cholestasis indicators, such as ALP and gamma-glutamyl transpeptidase, and the Toronto criteria (ALP > 1.67 × ULN) were not associated with LT-free survival, whereas the Paris-1, Paris-2, and Rotterdam criteria were found to predict prognosis. Multivariate analysis demonstrated that AST (HR, 1.796; 95% CI, 1.114-2.896; *P* = 0.016), ALB (HR, 0.005; 95% CI, 0.001-0.114; *P* = 0.001), TBIL (HR, 1.343; 95% CI, 1.151-1.568; *P* < 0.001), and serum creatinine (HR, 4.436; 95% CI, 2.082-9.451; *P* < 0.001) were associated with LT-free survival (Supplementary Table S2).

ALP and TBIL levels are important indicators of PBC prognosis. To further explore the prognostic value of ALP, we stratified the included population according to TBIL values of 1.0 × ULN, 1.5 mg/dL, and 2.0 mg/dL at baseline and at the 1-year follow-up. As shown in Supplementary Table S3, the HRs of univariate Cox regression analyses were all less than one when TBIL was greater than 1.0 × ULN, 1.5 mg/dL, or 2.0 mg/dL, suggesting that elevated ALP may be a risk factor. Conversely, the HRs were all less than one when TBIL was lower than 1.0 × ULN, 1.5 mg/dL, or 2.0 mg/dL, suggesting that elevated ALP may be a protective factor. This was especially evident when we stratified the enrolled population according to a TBIL value of 1.0 × ULN at the 1-year follow-up, as the P values of both groups were less than 0.1.

### Exploring the suitable recompensation criteria for PBC

Based on these findings, item A was defined as a biochemical response using the Paris-1, Paris-2, and Rotterdam criteria. We defined item B of recompensation as the absence of decompensating events from the last decompensated state in the following 12 months. ^[19]^ We defined item C of recompensation as MELD < 10 or Child-Pugh A.^[20]^ Given that ALP is a crucial marker for assessing biochemical response and considering the complex prognostic values of ALP in the enrolled patients, we also defined recompensation as items B and C.

Table 2 shows the effectiveness of various recompensation criteria in evaluating the primary outcomes. Compared to item A + item B + MELD < 10, the 3-year and 5-year areas under the receiver operating characteristic curves, as well as the C-index of item B + MELD < 10, were superior. Similarly, item B + Child-Pugh A showed the best results. The recompensation criteria, when combined with biochemical responses, did not enhance the predictive power of the primary outcomes defined by item B + MELD < 10 or item B + Child-Pugh A.

**Table 2:** Prediction efficacy of various recompensation criteria for the primary outcomes.

Characteristics	3-year AUC (95% CI)	*P* value	5-year AUC (95% CI)	*P* value	C-index (95% CI)
MELD <10 + B	0.675 (0.596- 0.755)		0.713 (0.630- 0.795)		0.651 (0.621- 0.681)
Paris 1 + MELD <10 + B	0.641 (0.595- 0.688)	0.344	0.676 (0.610- 0.743)	0.262	0.618 (0.598- 0.638)
Paris 2 + MELD <10 + B	0.598 (0.557- 0.639)	0.042	0.608 (0.551- 0.665)	0.262	0.580 (0.563- 0.597)
Rotterdam + MELD <10 + B	0.598 (0.557- 0.639)	0.042	0.618 (0.559- 0.676)	0.006	0.579 (0.562- 0.596)
Child-Pugh A + B	0.648 (0.569- 0.727)		0.693 (0.611- 0.775)		0.631 (0.602- 0.660)
Paris 1 + Child-Pugh A + B	0.630 (0.585- 0.676)	0.613	0.676 (0.610- 0.743)	0.578	0.611 (0.591- 0.631)
Paris 2 + Child-Pugh A + B	0.592 (0.553- 0.632)	0.133	0.608 (0.551- 0.665)	0.021	0.577 (0.560- 0.594)
Rotterdam + Child-Pugh A + B	0.598 (0.557- 0.639)	0.172	0.618 (0.559- 0.676)	0.037	0.579 (0.562- 0.596)
MELD <10 + B	0.675 (0.596- 0.755)		0.713 (0.630- 0.795)		0.651 (0.621- 0.681)
Child-Pugh A + B	0.648 (0.569- 0.727)	0.022	0.693 (0.611- 0.775)	0.153	0.631 (0.602- 0.660)
MELD <10 + B& Child-Pugh A + B	0.648 (0.569- 0.727)	0.022	0.693 (0.611- 0.775)	0.153	0.623 (0.594- 0.652)

*P* value was compared between MELD <10 + B and Paris 1 + MELD <10 + B, Paris 2 + MELD <10 + B or Rotterdam + MELD <10 + B through Delong test. CI, confidence interval; AUC, the area under the receiver operating characteristic curve; B, absence of decompensating events from the last decompensated state in the next 12 months.

Compared to item B + Child-Pugh A, item B + MELD < 10 improved the predictive power of the 3-year primary outcomes (Table 2), and the combination of item B + MELD < 10 and item B + Child-Pugh A did not improve the predictive power of the primary outcomes established by item B + MELD < 10.

Therefore, we defined recompensation as items B (absence of decompensating events from the last decompensated state in the following 12 months) and C (MELD score < 10) in patients with PBC-related decompensated cirrhosis.

### Recompensating status and prognosis

**Figure 2:**
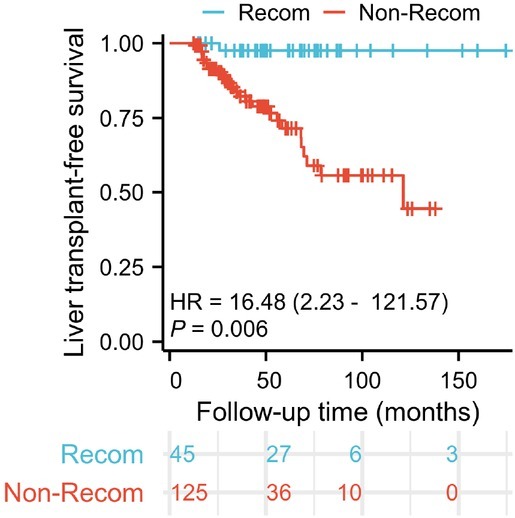
Kaplan-Meier plots of LT-free survival in patients with PBC-related decompensated cirrhosis, stratified by recompensation status. Recon, recompensation. LT: liver transplant; HR: hazard ratio.

Forty-five patients achieved recompensation at a median of 26 months (IQR, 23-37 months) after enrolment. The recompensation rate of the included patients was 26% (45/170). The primary and secondary outcome rates were 2% (1/45) and 22% (10/45) in the recompensation group and 22% (27/125) and 63% (79/125) in the nonrecompensation group, respectively. Significant differences were observed in primary and secondary outcome rates between the recompensation and non-recompensation groups (*P* < 0.05). Cox analyses revealed a substantial increase in the hazards of liver-related death or LT attributable to non-recompensation (HR, 16.48; 95% CI, 2.23-121.57; *P* = 0.006; Figure 2).

### Dynamic changes in risk stratification and serum biochemical indicators

**Figure 3:**
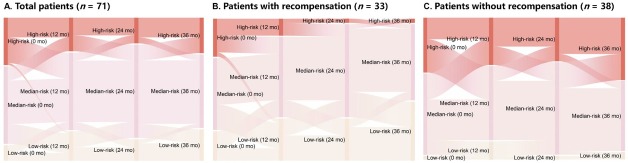
Sankey diagrams illustrating changes in risk stratification from baseline to 36 months. Sankey diagrams represent major transitions in patient risk status. Colors indicate different GLOBE classifications: yellow for low risk, pink for medium risk, and red for high risk. Column lengths represent patient proportions in each risk category. Thicker lines indicate a greater number of patients transitioning between categories. Patients with PBC were stratified as low, medium, or high risk based on GLOBE values of 0.5 and 1.8. (A) Total patients (*n* = 71). (B) Patients with recompensation (*n* = 33). (C) Patients without recompensation (*n* = 38).

**Figure 4:**
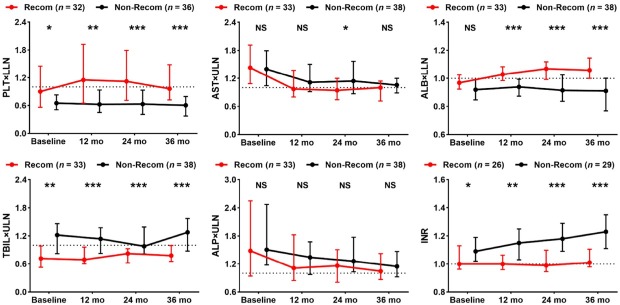
Dynamic changes in serum indicators in patients with and without recompensation over 36 months of treatment. Median values and IQRs are shown. Differences between groups at each time point were assessed using Mann-Whitney U tests. Recon, recompensation. PLT: platelet; AST: aspartate aminotransferase; ALB: albumin; TBIL: total bilirubin; ALP: alkaline phosphatase; INR: international normalized ratio; mo: months; ULN: upper limit of normal; LLN: lower limit of normal; IQR: interquartile range; NS: not significant. ^*^P < 0.05, ^**^P < 0.01, ^***^P < 0.001.

Our study cohort included 71 (42%) patients with concurrent 1-, 2-, and 3-year follow-up data. A previous study confirmed that using GLOBE values of 0.5 and 1.8 could stratify patients with PBC into low-, medium-, and high-risk groups. 22 Accordingly, we plotted Sankey diagrams to represent changes in risk stratification using GLOBE scores from baseline to 36 months. These diagrams depicted a remarkably greater reduction in the proportion of high-risk patients in the recompensation group than in the non-recompensation group (Figure 3, Supplementary Table S4). Although both groups exhibited improvements in ALP and AST levels during the 36-month treatment period, the recompensation group demonstrated a significantly higher proportion of stable improvements in PLT, ALB, TBIL, and international normalized ratio (INR) levels (Figure 4).

### Baseline indicators, one-year biochemical criteria, and other parameters related to recompensation

As shown in Supplementary Table S5, baseline indicators, including sex, age, PLT, ALB, TBIL, and prothrombin time (PT), were associated with recompensation. Multivariate analysis demonstrated that PLT (HR, 1.688; 95% CI, 1.184-2.349; *P* = 0.003) and TBIL (HR, 0.517; 95% CI, 0.306-0.873; *P* = 0.014) were independently associated with recompensation. At the 1-year follow-up, univariate analysis indicated that the Paris-1, Paris-2, and Rotterdam criteria were associated with recompensation. Multivariate analysis demonstrated that the Rotterdam criteria (HR, 2.433; 95% CI, 1.350-4.421; *P* = 0.003) were independently associated with recompensation.

A total of 106 (62%) patients underwent liver biopsy during the follow-up period, including 15 (14%) with severe interface hepatitis and seven (7%) with mild to moderate interface hepatitis. Chi-square test analysis revealed that neither mild to moderate interface hepatitis nor severe interface hepatitis was associated with recompensation (*P* = 0.067 and 0.313, respectively). However, Cox analysis revealed that severe interface hepatitis was associated with a decreased likelihood of recompensation (HR, 3.055; 95% CI, 1.261-7.399; *P* = 0.013).

## Discussion

This study highlights the complex prognostic implications of ALP in patients with PBC-related decompensated cirrhosis undergoing UDCA treatment. Specifically, high ALP levels in patients with normal TBIL were associated with unfavorable outcomes, whereas low ALP levels in patients with abnormal TBIL correlated with poor prognosis. A MELD score < 10 and the absence of decompensating events from the previous decompensated state within the following year serve as useful metrics for assessing PBC recompensation. Approximately 26% of the patients in this study experienced at least one episode of recompensation after UDCA treatment, which was associated with significant improvements in prognosis and serum biochemical indicators. Recompensation was linked to baseline PLT counts, TBIL levels, 1-year Rotterdam criteria, and severe interface hepatitis.

Few studies have provided practical insights into biochemical responses, risk assessment, and long-term prognosis for patients with PBC-induced decompensated cirrhosis receiving UDCA treatment. This knowledge gap largely stems from small study populations, limited treatment options, and the terminal disease stage. In addition to confirming a low biochemical response rate and poor prognosis, this study underscores the complex interplay between ALP levels and prognosis in this patient population. In patients with impaired liver function, ALP reduction accompanied by bilirubin elevation may resemble the “bile acid–transaminase separation” observed in severe hepatitis. This finding suggests that, in patients with abnormal TBIL levels, improving liver synthetic reserve function should be prioritized over solely focusing on etiology control and UDCA biochemical response. The same principle may apply to decompensated liver diseases of other aetiologies.

In the early decompensated stages (TBIL < 1), elevated ALP levels may reflect active biliary epithelial injury and compensatory regeneration of small bile ducts. Persistent ALP elevation indicates ongoing inflammation and fibrogenesis, which drive disease progression. Conversely, in advanced decompensated stages (TBIL > 1), severe cholestasis and hepatocyte failure predominate. Here, low ALP levels may indicate exhaustion of biliary repair mechanisms (including loss of functional bile ductules), leading to impaired ALP release and worse prognosis. Both bilirubin conjugates and ALP are excreted *via* bile. In advanced decompensated stages, dysfunction of canalicular transporters (including multidrug resistance-associated protein 2 [MRP2]) may trap bilirubin within hepatocytes, exacerbating toxicity. Paradoxically, reduced ALP levels in this context may reflect impaired biliary bicarbonate secretion (*via* ankylosis homolog (ANKH)*-*ALP interaction), which could exacerbate bile acid-mediated injury through acidic bile. Meanwhile, TBIL < 1 with high ALP levels may indicate incomplete compensation, where ALP elevation reflects retained adaptive responses (including alternative excretory pathways) despite ongoing autopathy.

Evaluating prognosis in patients with decompensated diseases presents a complex challenge. The BAVENO VII publication introduced a consensus definition of recompensation, which may provide valuable insights into risk assessment for this patient group. However, further work is required before these criteria can be applied to other diseases. In patients with PBC, defining suppression of the primary etiology of cirrhosis is a crucial first step. In this study, the biochemical response was established as an initial definition. However, the combined biochemical response did not improve predictive efficacy for prognosis in decompensated patients. The complex prognostic role of ALP may confound the utility of biochemical response criteria based on ALP in assessing recompensation. For simplicity, recompensation was defined as a MELD score < 10 and the absence of decompensating events for 12 months following the last decompensated state.

We further validated the recompensation criterion. The present study demonstrated that patients with PBC who achieved recompensation exhibited improved outcomes, including longer LT-free survival, lower rates of further decompensation, a significant reduction in the proportion of high-risk patients, and improved serum biochemical indicators during follow-up. These findings align with those reported for other liver diseases.^[19, 20, 21]^ Consequently, this definition of recompensation appears reasonable and could serve as an endpoint for clinical trials.

Although the multi-criteria integration initially aimed to comprehensively capture PBC progression and refine risk stratification, the added algorithmic complexity may compromise clinical utility. The prioritized simplified criteria (MELD < 10 and 12-month LT-free survival) adhere to Baveno VII’s priorities of feasibility and reproducibility, mirroring adaptations like spleen stiffness measurement (SSM) to streamline surveillance protocols.

Our previous study^[22]^ revealed that additional fenofibrate therapy was associated with high ALP normalisation rates and low UK-PBC risk scores^[23]^ in patients with cirrhotic PBC who exhibited an incomplete response to UDCA. Moreover, fenofibrate therapy appeared safe and well tolerated, with a low incidence of adverse effects in patients with cirrhosis. Given that fibrates were not recommended for decompensated patients, none of the patients in this study received fibrate therapy during follow-up. Exploring the use of fibrates in recompensated patients may present a promising approach to addressing limited treatment options.

Further decompensation refers to the development of a new complication related to a previous decompensating event or the occurrence of a second decompensating event with rapid, progressive clinical deterioration leading to liver transplantation or death.^[14]^ Both further decompensation and recompensation can serve as dynamic indicators for assessing prognosis in decompensated patients. Our recent research demonstrated that significant changes in liver stiffness could effectively predict prognosis in compensated patients with PBC.^[24]^ Future investigations should further explore the predictive value of liver stiffness and further decompensation in patients with PBC-related decompensated cirrhosis.

While this study focused on clinical and biochemical markers of recompensation, the absence of longitudinal liver stiffness measurements or histological data (*e.g*., ductopenia regression) precludes a direct evaluation of architectural recovery. Future studies incorporating noninvasive fibrosis monitoring (*e.g*., fibrosis 4 score (FIB-4), VCTE), paired liver biopsies, or advanced imaging (*e.g*., cT1 mapping) are pivotal to unraveling the histo-mechanistic basis of recompensation.

This study has several limitations, including its single-center retrospective design and relatively small sample size, which may limit the generalisability of findings and introduce potential selection bias. However, decompensated cirrhosis is an uncommon and advanced condition, making large-scale studies in real-world PBC populations challenging. Additionally, insufficient histological and liver stiffness data prevented a comprehensive evaluation of histological improvements following recompensation.

In conclusion, this study defined and validated a simplified definition of recompensation as MELD < 10 and the absence of decompensating events for 12 months following the last decompensated state in patients with PBC-related decompensated cirrhosis. These findings warrant further validation in larger, multicentre, long-term cohort studies.

## Supplementary Information

Supplementary materials are only available at the official site of the journal (www.intern-med.com).

## Supplementary Material

Supplementary Materials
