# Supplementary material for: Definition and validation of recompensation in patients with primary biliary cholangitis-related decompensated cirrhosis treated with ursodeoxycholic acid: Based on the BAVENO VII criteria
Source: J Transl Int Med. 2025 Jul 31;13(4):366–74. doi: 10.1515/jtim-2025-0034 (PMC12371399; doi:10.1515/jtim-2025-0034)
Supplement: Supplementary file 1 — Supplementary Materials [file jtim-2025-0034_sm.pdf]

Supplementary Table 1: Baseline parameters linked to the primary outcomes.

Characteristic	Univariate analysis			Multivariate analysis		
	***P* value**	**HR**	**95% CI**	***P* value**	**HR**	**95% CI**
Age (years)	0.003	1.070	1.022-1.119	0.007	1.063	1.017-1.112
Female sex	0.089	0.455	0.183-1.128			
ALP × ULN	0.832	1.030	0.787-1.348			
GGT × ULN	0.429	1.025	0.964-1.091			
PLT × LLN	0.842	1.057	0.610-1.832			
AST × ULN	0.068	1.500	0.971-2.319			
ALB × LLN	< 0.001	0.003	0.001-0.051	0.002	0.009	0.001-0.175
TBIL × ULN	< 0.001	1.499	1.206-1.864	0.001	1.503	1.191-1.898
Scr × ULN	0.084	6.360	0.780-51.839			
IgM × ULN	0.264	0.652	0.308-1.380			
INR	0.051	4.759	0.997-22.731			

Hazard ratios were calculated via the Cox regression model. ALP: alkaline phosphatase; GGT: gamma-glutamyl transpeptidase; PLT: platelet; AST: aspartate aminotransferase; ALB: albumin; TBIL: total bilirubin; Scr: serum creatinine; IgM: immunoglobulin M; INR: international normalised ratio; HR: hazard ratio; CI: confidence interval; ULN: upper limit of normal; LLN: lower limit of normal.

Supplementary Table 2: 1-year parameters linked to the primary outcomes

Characteristic	Univariate analysis			Multivariate analysis		
	***P* value**	**HR**	**95% CI**	***P* value**	**HR**	**95% CI**
Age (years)	0.001	1.083	1.034-1.133			
Female sex	0.089	0.455	0.183-1.128			
ALP × ULN	0.885	1.035	0.649-1.651			
GGT × ULN	0.350	1.074	0.925-1.247			
PLT × LLN	0.366	0.997	0.991-1.003			
AST × ULN	< 0.001	2.410	1.753-3.313	0.016	1.796	1.114-2.896
ALB × LLN	< 0.001	0.002	0.001-0.038	0.001	0.005	0.001-0.114
TBIL × ULN	< 0.001	1.433	1.274-1.611	< 0.001	1.343	1.151-1.568
Scr × ULN	0.009	2.830	1.303-6.145	< 0.001	4.436	2.082-9.451
IgM × ULN	0.417	1.197	0.776-1.846			
INR	0.008	2.133	1.218-3.736			
Paris 1	0.005	0.303	0.132-0.693			
Paris 2	0.004	0.118	0.028-0.499			
Rotterdam	0.025	0.193	0.046-0.812			
Toronto	0.492	1.351	0.573-3.182			

Hazard ratios were calculated via the Cox regression model. ALP: alkaline phosphatase; GGT: gamma-glutamyl transpeptidase; PLT: platelet count; AST: aspartate aminotransferase; ALB: albumin; TBIL: total bilirubin; Scr: serum creatinine; IgM:

immunoglobulin M; INR: international normalised ratio; HR: hazard ratio; CI: confidence interval; ULN: upper limit of normal; LLN: lower limit of normal.

Supplementary Table 3: Relationship between ALP and prognosis after stratification based on TBIL through univariate cox regression analysis

Parameters	***P* value**	**HR**	**95% CI**
TBIL levels at baseline			
< 1 × ULN	0.690	1.150	0.578-2.286
> 1 × ULN	0.537	0.908	0.668-1.234
< 1.5 mg/dl	0.270	1.277	0.827-1.971
> 1.5 mg/dl	0.128	0.744	0.508-1.089
< 2.0 mg/dl	0.320	1.199	0.838-1.717
> 2.0 mg/dl	0.120	0.703	0.450-1.097
TBIL levels at 1 year follow-up			
< 1 × ULN	0.025	2.567	1.123-5.866
> 1 × ULN	0.082	0.532	0.261-1.084
< 1.5 mg/dl	0.113	1.753	0.875-3.511
> 1.5 mg/dl	0.121	0.546	0.254-1.173
< 2.0 mg/dl	0.452	1.288	0.666-2.489
> 2.0 mg/dl	0.180	0.599	0.284-1.266

Hazard ratios were calculated via the Cox regression model. TBIL: total bilirubin; ALP: alkaline phosphatase; HR: hazard ratio; CI: confidence interval; ULN: upper limit of normal.

Supplementary Table 4: Risk stratified distribution of the standardized follow-up population at different time points according to the recompensation criteria defined by MELD <10 + B.

Characteristic	Total patients (*n* = 71)			Recompensation (*n* = 33)			Non-Recompensation (*n* = 38)			*P* value	
	High	Median	Low	High	Median	Low	High	Median	Low	Total	High-risk
0 month	24 (34)	39 (55)	8 (11)	9 (27)	17 (52)	7 (21)	15 (39）	22 (58)	1 (3)	0.042	0.278
12 months	17 (24)	39 (55)	15 (21)	4 (12)	19 (58)	10 (30)	13 (34)	20 (53)	5 (13)	0.047	0.030
24 months	13 (18)	47 (66)	11 (16)	1 (3)	22 (64)	10 (33)	12 (32)	25 (66)	1 (2)	<0.001	0.002
36 months	18 (25)	37 (52)	16 (23)	1 (3)	18 (54)	14 (43)	17 (45)	19 (50)	2 (5)	<0.001	<0.001

P value was compared between the recompensation and non-recompensation groups using the chi-squared test. B, absence of decompensating events from the last decompensated state in the next 12 months.

Supplementary Table 5: Baseline indicators and 1-year biochemical criteria between patients with and without recompensation

**Characteristics**	**Total (*n* = 170)**	**Recompensation (*n* = 45, 26%)**	**Non-Recompensation (*n* = 125, 74%)**	***P* value**
Baseline indicators				
Age (years)	57 ± 9	58 ± 9	54 ± 8	0.030
Female (*n*, %)	151(89)	107(86)	44(98)	0.026
Follow-up time (months)	45 ± 32	41 ± 30	54 ± 37	0.167
ALP × ULN	1.52(0.96-2.47)	1.53(0.98-2.43)	1.50(0.94-2.55)	0.872
PLT × LLN	0.72(0.57-1.03)	0.70(0.57-0.95)	0.90(0.59-1.42)	0.022
AST × ULN	1.43(1.06-2.08)	1.43(1.03-2.11)	1.43(1.09-1.94)	1.000
TBIL × ULN	1.09(0.75-1.70)	1.25(0.84-1.73)	0.75(0.55-1.25)	< 0.001
ALB × LLN	0.91(0.83-1.00)	0.90(0.81-0.97)	0.96(0.91-1.03)	0.004
Scr × ULN	0.69(0.58-0.83)	0.69(0.54-0.83)	0.72(0.63-0.84)	0.201
PT	1.06(1.00-1.16)	1.07(1.01-1.19)	1.01(0.96-1.11)	0.003
Treat-native	78(46)	25(56)	53(42)	0.129
**1-year criteria** (*n*, %)				
Paris 1	82(48)	47(38)	35(78)	< 0.001
Paris 2	57(34)	32(26)	25(56)	< 0.001
Rotterdam	42(25)	19(15)	23(51)	< 0.001
Toronto	119(70)	86(69)	33(73)	0.569

Continuous variables were expressed as mean ± SD or median (interquartile range), while categorical variables were presented as *n* (%). Comparisons between groups were made via the Fisher exact probability test, chi-square test, Student-*t* test, or Mann-Whitney *U* test. ALP: alkaline phosphatase; PLT: platelet count; AST: aspartate aminotransferase; ALB: albumin; TBIL: total bilirubin; Scr: serum creatinine; INR: international normalised ratio; PT: prothrombin time; ULN: upper limit of normal; LLN: lower limit of normal.
